# Increased alpha-defensin expression is associated with risk of coronary heart disease: a feasible predictive inflammatory biomarker of coronary heart disease in hyperlipidemia patients

**DOI:** 10.1186/s12944-016-0285-5

**Published:** 2016-07-18

**Authors:** Yaowapa Maneerat, Kriengchai Prasongsukarn, Surachet Benjathummarak, Wilanee Dechkhajorn, Urai Chaisri

**Affiliations:** Department of Tropical Pathology, Faculty of Tropical Medicine, Mahidol University, Bangkok, 10400 Thailand; Pramongkutklao Hospital and College of Medicine, Bangkok, 10400 Thailand; Center of Excellence for Antibody Research, Faculty of Tropical Medicine, Mahidol University, Bangkok, 10400 Thailand

**Keywords:** alpha-defensin, HNP 1–3, Neutrophil, CHD, Coronary heart disease, Hyperlipidemia, Biomarker

## Abstract

**Background:**

Atherosclerosis is a multifactorial disorder of the heart vessels that develops over decades, coupling inflammatory mechanisms and elevated total cholesterol levels under the influence of genetic and environmental factors. Without effective intervention, atherosclerosis consequently causes coronary heart disease (CHD), which leads to increased risk of sudden death.

Polymorphonuclear neutrophils play a pivotal role in inflammation and atherogenesis. Human neutrophil peptides (HNPs) or alpha (α)-defensins are cysteine-rich cation polypeptides that are produced and released from activated polymorphonuclear neutrophil granules during septic inflammation and acute coronary vascular disorders. HNPs cause endothelial cell dysfunction during early atherogenesis. In this cross-sectional study, control, hyperlipidemia and CHD groups were representative as atherosclerosis development and CHD complications. We aimed to assess the correlation between α-defensin expression and the development of CHD, and whether it was a useful predictive indicator for CHD risk.

**Methods:**

First, DNA microarray analysis was performed on peripheral blood mononuclear cells (PBMCs) from Thai control, hyperlipidemia and CHD male patients (*n* = 7). Gene expression profiling revealed eight up-regulated genes common between hyperlipidemia and CHD patients, but not controls. We sought to verify and compare α-defensin expression among the groups using: 1) real-time quantitative RT-PCR (qRT-PCR) to determine α-defensin mRNA expression (*n* = 10), and 2) enzyme-linked immunosorbent assay to determine plasma HNP 1–3 levels (*n* = 17). Statistically significant differences and correlations between groups were determined by the Mann–Whitney *U* test or the Kruskal–Wallis test, and the Rho-Spearman correlation, respectively.

**Results:**

We found that α-defensin mRNA expression increased (mean 2-fold change) in the hyperlipidemia (*p* = 0.043) and CHD patients (*p* = 0.05) compared with the controls. CHD development moderately correlated with α-defensin mRNA expression (*r* = 0.429, *p* = 0.023) and with plasma HNP 1–3 levels (*r* = 0.486, *p* = 0.000).

**Conclusions:**

Increased α-defensin expression is a potential inflammatory marker that may predict the risk of CHD development in Thai hyperlipidemia patients.

## Background

Atherosclerosis is a multifactorial disorder of heart blood vessels that develops over decades, coupling inflammatory mechanisms and dyslipidemia, and resulting in the formation of atherosclerotic plaques. Atherosclerosis develops under the influence of genetic and environmental factors. It is a chronic inflammatory disorder of the vessel wall, where both innate and adaptive immune responses influence disease progression [1]. This involves: impairment of endothelial cell (EC) function; accumulation of cholesterol in subendothelial macrophage-derived foam cells; adherence and recruitment of leukocytes into the arterial wall; proliferation and migration of smooth muscle cells into the intima; activation and aggregation of platelets; T cell activation; and production of inflammatory cytokines [2]. The innate signals within the lesion can arise from various sources and promote atherosclerosis through inflammatory processes. Oxidized low-density lipoprotein (LDL) accumulation, starting in the fatty streaks, promotes the inflammatory response, which most likely continues throughout lesion development. Furthermore, pathogenic infection and endogenous danger signals increase during tissue injury and have been implicated as inducers of lesion inflammation. ECs are activated by accumulation of modified LDL and increase the expression of adhesion molecules, mainly vascular cell adhesion molecule (VCAM-1) [3]. EC activation occurs predominantly at atherosclerosis-prone sites with increased hemodynamic strain, leading to infiltration of monocytes and T cells into the arterial wall [4, 5]. The recruitment of immune cells is a critical early step in atherosclerosis development and is potentiated by local chemokine release. Monocyte chemotactic protein-1 (MCP-1) appears to be particularly essential for attracting monocytes, whereas T helper type 1 (Th1) cells are recruited to the lesion by production of regulated upon activation, normal T cell expressed and secreted (RANTES), interferon inducible protein (IP)-10 and interferon-inducible T cell alpha chemoattractant (I-TAC) [6–9]. The infiltrated cells further increase local inflammation through production of several cytokines that increase recruitment and activation of additional immune cells. Monocytes differentiate into macrophages in the presence of macrophage colony-stimulating factor (M-CSF) produced in lesions by ECs and smooth muscle cells. This is a crucial step in atherogenesis and is accompanied by up-regulation of innate immune receptors required for phagocytosis and induction of inflammatory processes [6].

Moreover, previous studies have revealed that polymorphonuclear neutrophils (PMNs) play a prominent inflammatory role in atherogenesis in humans [7], mice [8] and pigs [9]. An earlier study has found PMNs in human tissues at lesions of plaque rupture and erosion, or in thrombi from acute coronary syndrome patients [10]. Previous studies have hypothesized that the number of PMNs in circulation, and the amount of PMN-produced elastase and myeloperoxidase (MPO) correlate with atherosclerosis [10, 11]. A positive correlation between peripheral PMN density and myocardial infarction has also been consistently observed [12].

Earlier studies have reported that human neutrophil peptides (HNPs) play a role in endothelial cell dysfunction during early atherosclerotic development. HNPs are cysteine-rich positively charged polypeptides that are produced and released from activated PMN granules. The α-defensin genes, DEFA1 and DEFA3, encode HNP-1, 2 and 3 [13, 14]. During inflammation, large amounts of intracellular proteins are released from activated PMNs into the extracellular milieu as a consequence of PMN degranulation, leakage during phagosome formation, and cell death. The highly homologous HNP-1, -2 and -3 make up more than half of the total protein contents within PMN azurophilic granules [15]. Moreover, HNP 1–3 levels are markedly increased in inflammation, including sepsis and acute coronary vascular disorders [1].

The present study was a cross-sectional design, with three male groups, including healthy control subjects, patients with hyperlipidemia and patients with CHD. These groups were representative of atherosclerosis development and CHD complications. Our data demonstrated an association between α-defensin expression levels and CHD development. We suggest that this knowledge may be applied to develop a key inflammatory marker for CHD risk in Thai hyperlipidemia patients.

## Methods

### Materials

In this study we used D-PBS (Wisent Inc., Quebec, Canada), TRIzol^®^ Reagent (Invitrogen™, Carlsbad, CA, USA), IsoPrep (Robbins Scientific Corporation, Sunnyvale, CA, USA), RNeasy total RNA kit (Qiagen, Hilden, Germany), and the Affymetrix GeneChip® Human Gene 1.0 ST Arrays (Affymetrix, Santa Clara, CA, USA). For qRT-PCR, we designed primers based on previous studies and Genbank, and had primers synthesized by Pacific Science Co., Ltd. (Bangkok, Thailand). Human HNP 1–3 enzyme-linked immunosorbent assay (ELISA) reagents were purchased from Hycult^®^ Biotech (Uden, The Netherlands). All other reagents were from Sigma-Aldrich (St. Louis, MO, USA).

### Study design and patient population

**Fig. 1:**
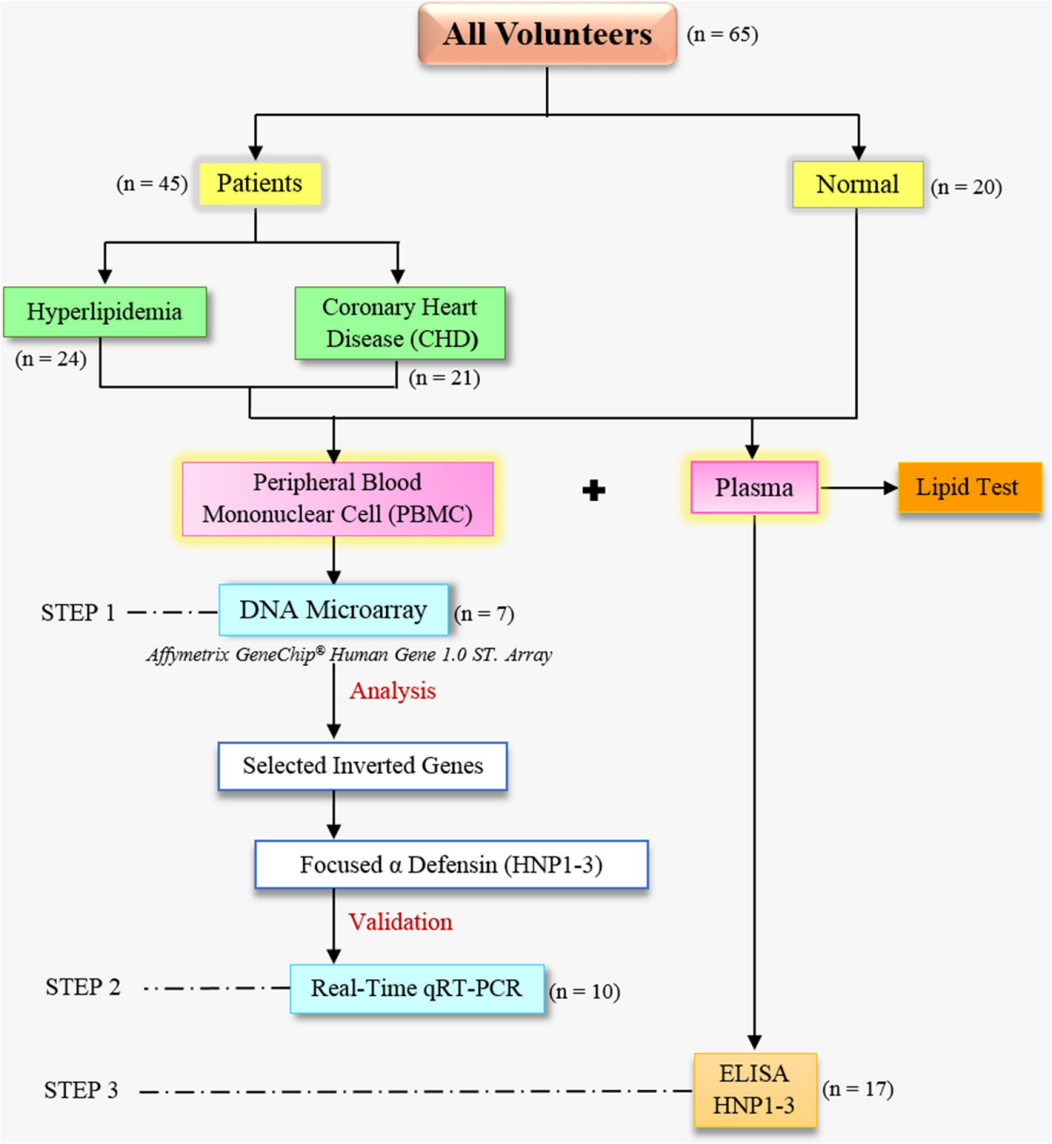
Experimental design and study population

The study design and characteristics of all volunteers enrolled in the study are summarized in Fig. 1. The study was conducted in the Department of Tropical Medicine, Mahidol University. Approval for the study was obtained from the Ethics Committees of Faculty of Tropical Medicine, Mahidol University, and Pramongkutklao Hospital. All participants were informed of the objectives of the study and completed an informed consent form.

All volunteers were unrelated males and born to Thai parents. All patients were diagnosed, classified and treated by a specialist (KP) at Pramongkutklao Hospital. Here, we focused our study on two groups of patients based on their clinical outcomes according to the criteria of the American Association. The 45 patients examined included: 1) 24 hyperlipidemia patients with high cholesterol levels [total cholesterol (TC), LDL and high-density lipoprotein (HDL)], but with no evidence of vital organ dysfunction; and 2) 21 CHD patients who had been diagnosed and had coronary bypass grafting under KP supervision. An additional 20 healthy controls were non-infected, and had no underlying disease or cardiovascular risk factors. The age distribution did not significantly differ between the groups. Before inclusion, all healthy volunteers and hyperlipidemia patients had not received any cholesterol or blood pressure-lowering medication. CHD patients were enrolled before coronary bypass grafting by KP.

### Blood sample collection

Heparinized blood samples (5 mL) were collected once from healthy donors and from all patients before hyperlipidemia treatment or coronary bypass grafting. Plasma (2 mL) was immediately collected by centrifugation of whole blood, and an aliquot for lipid measurement was kept at −70 °C for detection of HNP 1–3 by ELISA (Hycult Biotech). Packed blood cells were resuspended in D-PBS (Wisent Inc.) and used to isolate mononuclear cells. Approximately 2 million peripheral blood mononuclear cells (PBMCs) in TRIzol (Invitrogen™) were kept at −70 °C for gene expression profiling by DNA microarray analysis using Affymetrix GeneChip® Human Gene 1.0 ST. α-defensin expression was validated by qRT-PCR.

### Lipid measurement

Lipid markers including TC, TG, HDL cholesterol (HDL-c) and LDL cholesterol (LDL-c) were analyzed enzymatically using kits (Randox Laboratories limited, Crumlin, UK) and a biochemistry analyzer (Architect CI 16200, Abbott Laboratories, Abbott Park, IL, USA).

### Mononuclear cell isolation

PBMCs were separated by Isoprep (Robbins Scientific Corporation) gradient centrifugation according to the manufacturer’s recommendation.

### PBMC gene expression profiling using DNA microarray analysis

Total RNA was extracted from PBMCs of hyperlipidemia patients and CHD patients who had undergone bypass grafting, and of control volunteers (*n* = 3) using RNA isolation kits (Qiagen). Total RNA was quantified using a NanoDrop ND-1000 spectrophotometer with ND-1000 3.3 software, and RNA integrity (RIN) was examined using an Agilent Bioanalyzer (Santa Clara, CA, USA). Affymetrix GeneChip® Human Gene 1.0 ST was performed using 5 μg of total RNA with RIN ≥ 8.0, according to the manufacturer’s protocol (Affymetrix Inc.). The data were analyzed by Agilent GeneSpring GX Software version 12.0. Differentially expressed genes correlating with inflammation were identified using the criteria of a > 2.0-fold increase/decrease expression in the two patient groups compared with the control group [7]. Up-regulated genes common between hyperlipidemia and CHD patients, but not with controls were selected to further assess the feasibility of using them as inflammatory markers of CHD development [16].

### Validation of α-defensin DEFA1/DEFA3 expression

#### Investigation of α-defensin mRNA expression by qRT-PCR

Total RNA was isolated from PBMCs (2 × 10^6^) using Trizol (Invitrogen). cDNA was synthesized using 1 μg of total RNA with SuperScript III First-Strand Synthesis System for RT-PCR (Invitrogen), according to the manufacturer’s protocol. We designed primers based on DEFA1/DEFA3 genes (GenBank accession numbers NM_005217.3). qRT-PCR was performed in duplicate. Each 20-μl PCR reaction contained 10 μl of LightCyCler 480 SYBR Green I Master mix (Roche Diagnostic, Mannheim, Germany) mixed with 100 ng of cDNA and 0.5 μM of each primer (forward: 5′-TCCTTGCTGCCATTCTCCTG-3′ and reverse: 5′-TGCACGCTGGTATTCTGCAA-3′). Amplification was conducted in a LightCycler® Real-Time PCR system (Roche Applied Science, Indianapolis, IN, USA). PCR reactions were subjected to 95 °C for 5 min, followed by 45 cycles of denaturation at 95 °C for 30 s, annealing at 60 °C for 30 s, and melting curve analysis at 65 °C for 1 min. The expected PCR product size was 204 bp. β-Actin (ACTB) primers (forward: 5′-TCACCCACACTGTGCCCATCTACGA −3′ and reverse: 5′ -CAGCGGAACCGCTCATTGCCAATGG-3′) were used to normalize the relative expression level of α-defensin [17]. The 2^−(ΔΔCt)^ method was used to quantify relative expression levels.

#### Determination of plasma HNP 1–3 by ELISA

Plasma was centrifuged at 2500 × g for 5 min and HNP 1–3 concentrations were measured by ELISA (Hycult Biotechnology), according to the manufacturer’s instructions. Briefly, 100 μl of plasma from the three groups (*n* = 17) were diluted 20-fold in sample dilution buffer, and standards at concentrations of 10,000 to 136 pg/ml were transferred to microtiter wells coated with the specific antibody and incubated for 60 min at room temperature (RT). Each well was then washed to remove unbound material and biotinylated tracer antibody (100 μl) was added, followed by incubation for 60 min at RT. After removing unbound materials, streptavidin-peroxidase conjugate was added (100 μl/well) and incubated for 60 min at RT. Bound enzyme was detected by adding 100 μl of tetramethylbenzidine substrate to each well, and reactions were stopped by adding 100 μl of stop solution. Optical density was determined at 450 nm using Tecan Sunrise OEM Remote Microplate Absorbance Reader (Tecan, Grödig, Austria). HNP 1–3 concentrations were calculated from the HNP 1–3 standard curves.

### Statistical analysis

Clinical data are reported as median (upper and lower range limits). The amount of α-defensin mRNA is represented as fold change relative to healthy controls. HNP 1–3 levels were expressed as median ± SEM. The significance of the differences between two groups was determined by the Mann–Whitney *U* test, and those among three groups were determined by the Kruskal–Wallis test. Correlations between CHD development and α-defensin mRNA expression or plasma HNP 1–3 levels were analyzed by the Rho-Spearman correlation analysis. The α level was set at < 0.05 at a 95 % confidence interval. All statistical analyses were performed using SPSS version 11.5 (SPSS, Chicago, IL, USA).

## Results

### Clinical manifestations

The baseline characteristics of all patients and healthy controls are summarized in Table 1. The healthy and hyperlipidemia patients did not differ in age, whereas the CHD patients were significantly older compared with the control and hyperlipidemia groups (both *p* = 0.000). Hyperlipidemia patient TC levels were significantly higher than those in control (*p* = 0.003) and CHD patients (*p* = 0.007). The TC, LDL-c and HDL-c levels of the CHD group were not significantly different from those of the controls (*p* > 0.050). However, the hyperlipidemia patient LDL levels were higher than those in CHD patients (*p* = 0.039).

**Table 1:** General description and clinical manifestations of the study population

Variables	Normal (N) *n* = 17	Hyperlipidemia (H) *n* = 17	Coronary Heart Disease (CHD) *n* = 17	*p* values		
				N vs. H	H vs. CHD	N vs. CHD
Age (years)	42 (23–58)	42 (26–58)	66 (58–78)	0.608	**0.000**	**0.000**
TC (mg/dL)	174.5 (156–199)	223 (150–304)	166 (115–259)	**0.004**	**0.008**	0.653
TG (mg/dL)	147 (70–162)	166 (103–1181)	92 (72–169)	0.759	**0.013**	**0.025**
HDL (mg/dL)	41 (31–56)	45.5 (26–80)	49 (37–75)	0.213	0.496	0.063
LDL (mg/dL)	99 (60–111)	130.5 (63–190)	89 (44–174)	0.072	**0.049**	0.323

All patients and controls were male. *N* normal controls, *H* and *CHD* patients with hyperlipidemia and coronary heart disease, respectively

Data are shown as medians (ranges). The differences in each variable between two groups (N vs. H, H vs. CHD, and N vs. CHD) were determined using the Mann–Whitney *U* test. The α level was set at < 0.05 at a 95 % confidence interval. The significantly different variables between groups are shown by *p* value in bold

*TC* total cholesterol, *TG* triglyceride, *HDL* high-density lipoprotein, *LDL* low-density lipoprotein

### Profiling of hyperlipidemia and CHD patients reveals common up-regulated genes

**Fig. 2:**
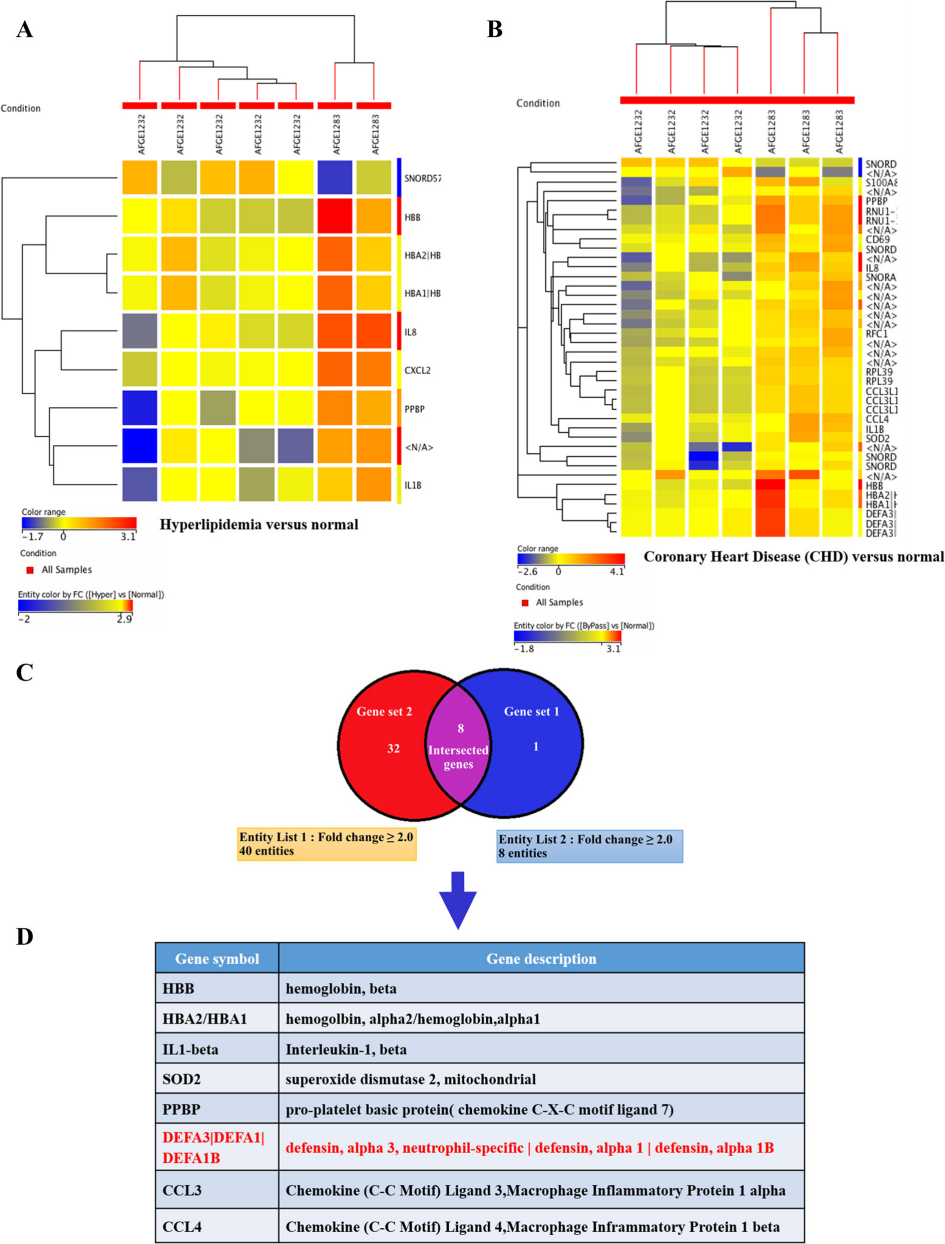
Gene expression profiling by DNA microarrays. **a** and **b** Heat maps of differentially expressed transcripts in peripheral blood mononuclear cells (PBMCs) from coronary heart disease (CHD) patients post coronary bypass grafting vs. control **a** and hyperlipidemia patients vs. control **b**. Total RNA was extracted from 2 million PBMCs (*n* = 7). Differentially expressed genes > 2.0-fold were further evaluated: **c** Venn diagram illustrating the eight genes up-regulated in the two patient groups; **d** List of the eight genes common to both patient groups

We observed nine up-regulated genes (entity list 2) in the hyperlipidemia group and 40 genes (entity list 1) in the CHD group (Fig. 2) relative to the baseline by DNA microarray analysis. Of these, eight genes were common to both groups of patients (Fig. 1c and d, and Table 2). Based on these results, we sought to validate α-defensin (one of eight intersected genes) expression and its association with CHD development.

**Table 2:** Profiles of intersected up-regulated genes in hyperlipidemia and CHD patients

**Gene symbol**	**Gene description**
***HBB***	hemoglobin beta
***HBA2***/***HBA1***	hemoglobin alpha2/hemoglobin alpha1
***IL*** *-* ***1-beta***	Interleukin-1 beta
***SOD2***	superoxide dismutase 2, mitochondrial
***PPBP***	pro-platelet basic protein (chemokine C-X-C motif ligand 7)
***DEFA3*** **|** ***DEFA1*** **|** ***DEFA1B***	**defensin alpha 3, neutrophil-specific | defensin alpha 1 | defensin alpha 1B**
***CCL3***	chemokine (C-C motif) ligand 3, macrophage inflammatory protein 1 alpha
***CCL4***	chemokine (C-C motif) ligand 4, macrophage inflammatory protein 1 beta

The gene in bold was selected as a focus that was validated using qRT-PCR and ELISA

### Increased expression of α-defensin mRNA in hyperlipidemia and CHD patients

**Fig. 3:**
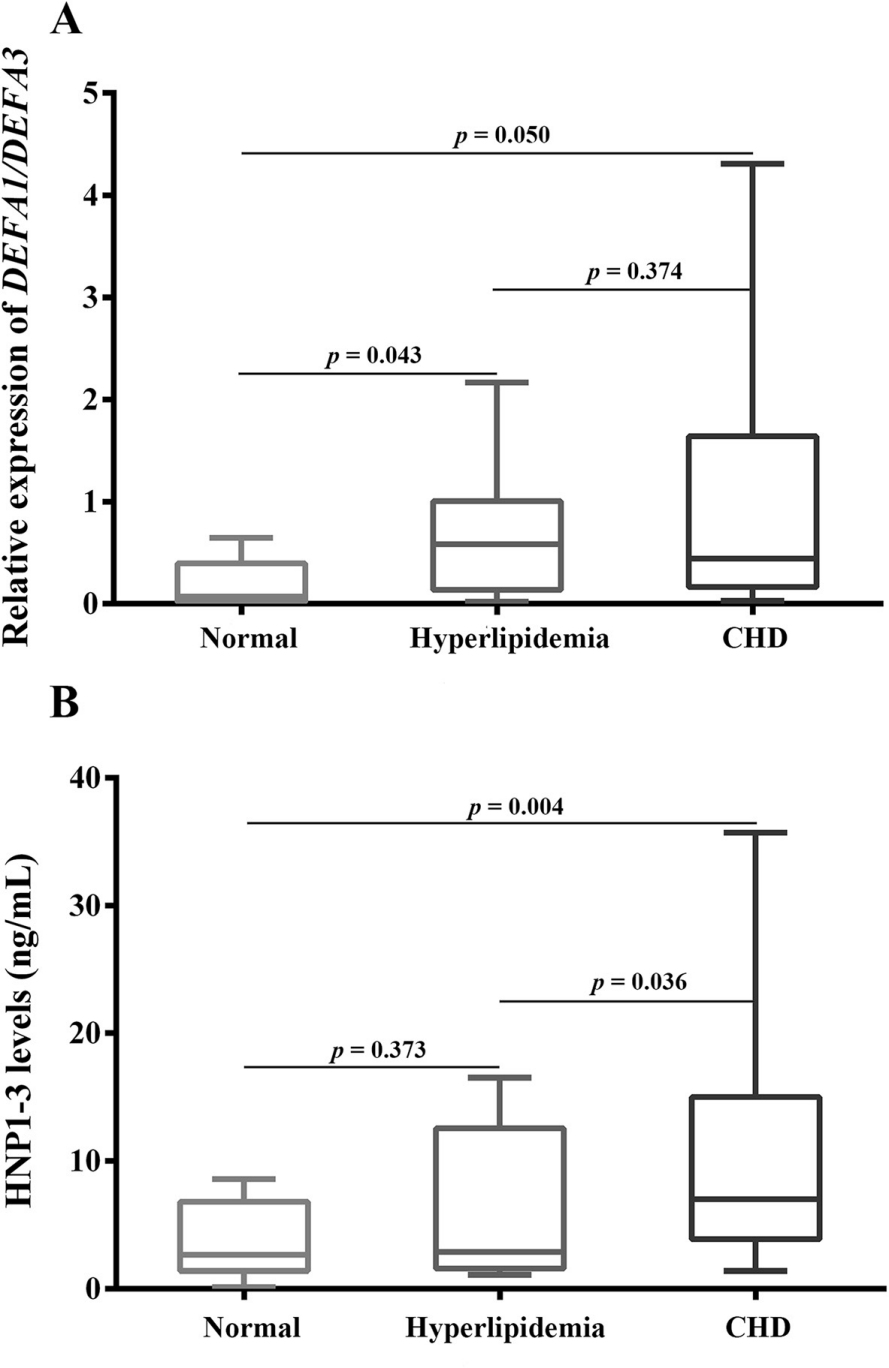
Expression of α-defensin and HNP 1–3. **a** α-Defensin mRNA expression (2-fold changes) relative to β-actin mRNA in PBMCs obtained from healthy controls, and hyperlipidemia and CHD patients post graft bypass surgery, as determined by qRT-PCR. Data are presented as the mean 2-fold change relative to control ± SEM (*n* = 10). **b** Plasma levels of HNP 1–3 (pg/ml) from healthy controls, hyperlipidemia patients and CHD patients post graft bypass surgery. Data are presented as mean ± SEM (*n* = 17). One-way ANOVA followed by Scheffe’s *post hoc* test for statistical significance of mRNA expression and HNP levels (*p* < 0.05) were used for statistical comparisons

The relative expression levels of α-defensin mRNA in healthy, hyperlipidemia and CHD patients are shown in Fig. 3a. Our findings revealed that α-defensin mRNA expression (mean 2-fold change) in hyperlipidemia (*p* = 0.043) and bypass graft surgery (*p* = 0.05) patients was increased compared with controls. Notably, the relative expression differences between CHD and hyperlipidemia patients were not significant (*p* = 0.374).

### Increased levels of plasma HNP 1–3 in hyperlipidemia and CHD patients

There was a trend of increased plasma HNP 1–3 in hyperlipidemia (*p* = 0.373) and CHD patients (*p* = 0.004) compared with the levels in the healthy volunteers. Furthermore, CHD patients had higher HNP 1–3 levels than hyperlipidemia patients (*p* = 0.036; Fig. 3b).

### Correlations between clinical manifestation, expression of DEFA1/DEFA3, HNP 1–3 levels and CHD development

**Fig. 4:**
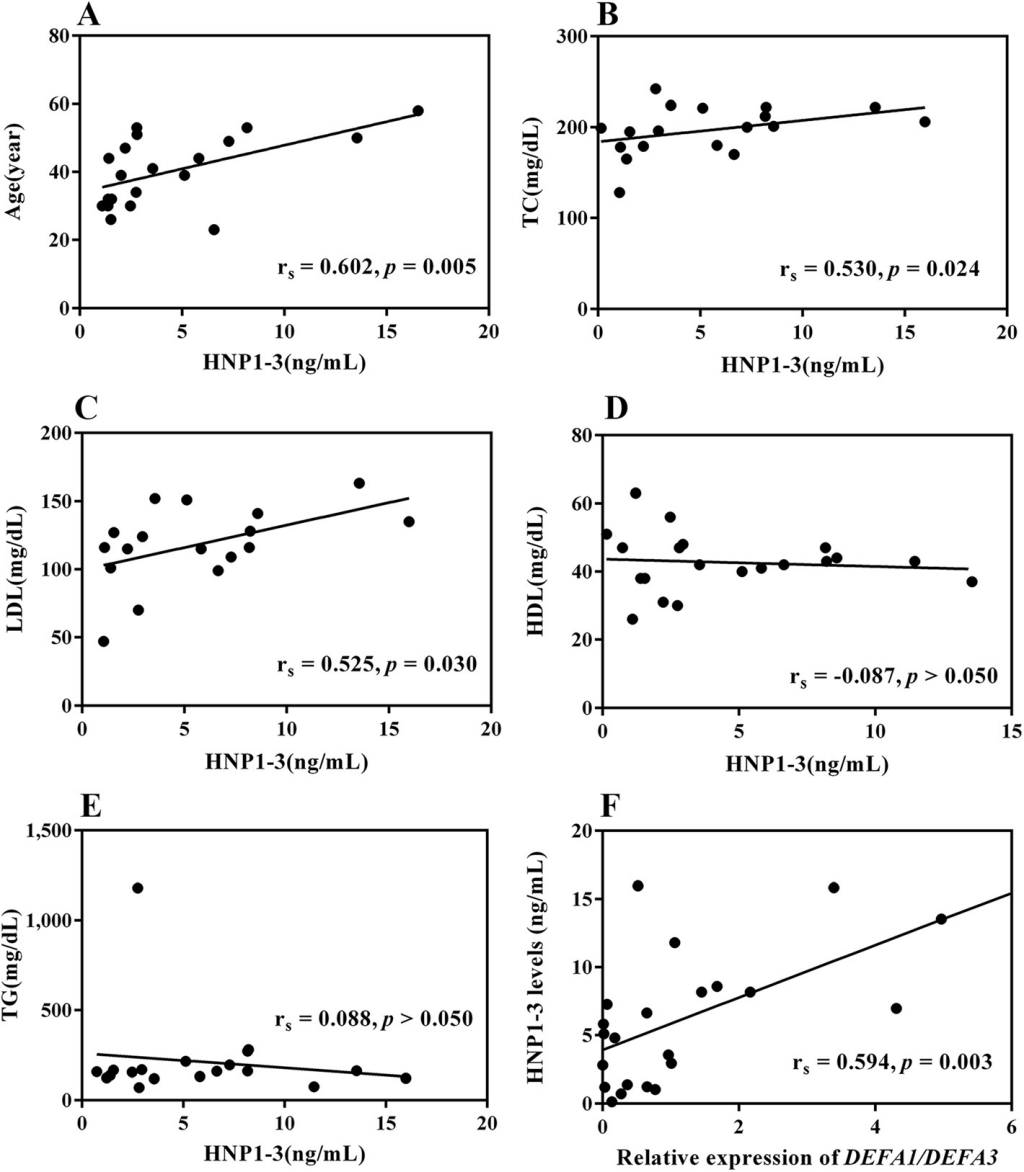
Correlations between plasma HNP 1–3 levels and age **a** TC **b** LDL **c** HDL **d** and TG **e** and correlation between relative expression of DEFA1/DEFA3 and HNP 1–3 levels **f**. *Note*: Results in **a** and **f** were analyzed among the three groups; in **b**–**e** patients with CHD were excluded, because they used lipid-lowering drugs

Our cross-sectional study assumed that pathologic changes progress from normal to hyperlipidemia and finally to CHD complications. The correlations are shown in Fig. 4. There was a moderate correlation between DEFA1/DEFA3 gene expression and HNP plasma levels (*r* = 0.536, *p* = 0.010). Additionally, α-defensin DEFA1/DEFA3 mRNA expression positively correlated with CHD development (*r* = 0.429, *p* = 0.023). Elevated plasma HNP 1–3 levels were consistently associated with CHD development (*r* = 0.486, *p* = 0.000), and CHD correlated with age (*r* = 0.602, *p* = 0.005). Lipid profiling revealed a correlation between plasma HNP 1–3 levels in normal and hyperlipidemia patients as indicated by TC (*r* = 0.530, *p* = 0.024) and LDL (*r* = 0.525 *p* = 0.030), but not by HDL (*r* = −0.870, *p* = 0.714) and TG (r = 0.088, *p* = 0.721) levels.

## Discussion

Our cross-sectional study revealed that CHD development is associated with an increase in DEFA1/DEFA3 mRNA expression and plasma HNP 1–3 levels. Based on these results, we suggest that increased α-defensin expression has potential as an inflammatory marker for predicting CHD in hyperlipidemia patients. However, α-defensin expression as a predictive marker should be confirmed on a larger patient cohort in a future multicenter study, to validate these observations.

Previous cross-sectional studies covered a shorter observation time and were more appropriate for understanding the long-term development from initiation to complete pathogenesis [18–22]. Similarly, we profiled the expression of control, hyperlipidemia and CHD groups that are representative of the development of atherosclerosis to CHD (Fig 1).

In this preliminary study, we chose only male volunteers to control the influence of the sex-hormone factor. Estrogens are primary examples of female sex steroids. Epidemiological studies in animal models, e.g., rabbits, mice, and monkeys, have shown that estrogen has protective effects in cardiovascular disease. Previous evidence has also suggested that estrogen protects women against CHD pre-menopause (reviewed in [23]). We expect that the results of this study will help further studies find appropriate biomarkers to predict CHD in both male and female Thai hyperlipidemia patients.

Blood is an accessible source for diagnosing disease processes in disorders such as autoimmune disease, asthma and breast cancer [24]. Blood is a particularly fitting surrogate for atherosclerotic tissue, because it contains inflammatory cells, which play an important role in atherogenesis [1]. Consistent with this knowledge, previous studies used PBMC expression profiling to study the pathogenesis, diagnosis and pharmacokinetics in human atherosclerosis, stroke and other vascular diseases [24–26]. These approaches are consistent with our study, which used PBMCs from healthy, hyperlipidemia and CHD patients to investigate expression differences associated with atherosclerosis and CHD complications.

In this study, we designed the DEFA1/DEFA3 qRT-PCR primers based on DEFA1/DEFA3 genes (GenBank accession numbers NM_005217.3) and examined primers and target using primer blast from the NCBI website. The primer blast analysis showed that the DEFA1/DEFA3 primers in our study completely matched (100 %) the DEFA1/DEFA3 genes (NM_005217.3) and other variant defensin alpha genes in the NCBI database: 1. Homo sapiens defensin, alpha 3, neutrophil-specific (DEFA3), transcript variant X1, mRNA (XM_011534741.1), 2. Homo sapiens defensin, alpha 1 (DEFA1), transcript variant X3, mRNA (XM_011534740.1), 3. Homo sapiens defensin alpha 1B (DEFA1B), transcript variant 1, mRNA (NM_001302265.1), 4. Homo sapiens defensin alpha 1 (DEFA1), mRNA (NM_004084.3), and 5. Homo sapiens defensin alpha 1B (DEFA1B), transcript variant 2, mRNA (NM_001042500.1)

The PCR product size of this gene was 204 bp, as calculated by this program. Our DEFA1/DEFA3 primers were optimized and the size of target product determined by conventional PCR method and gel electrophoresis, respectively. We found a single band of PCR product of about 200 bp. In addition, all PCR products from qRT-PCR, which amplified with the DEFA1/DEFA3 qRT-PCR primers, were confirmed by gel electrophoresis. Those PCR products showed the same size as expected. The DEFA1/DEFA3 qRT-PCR primers were predicted to hit homo sapiens DEAH-box helicase 8 (DHX8) gene. However, both forward and reverse primers did not completely match the DHX8 sequence, and the PCR product was clearly different from the expected target product (660 bp). Therefore, our DEFA1/DEFA3 gene primer is specific enough and appropriate for qRT-PCR in this study.

For beta actin (ACTB) primers, we followed the primer sequences from previous studies [17, 27]. Using primer blast from the NCBI website, this ACTB primer set was predicted to hybridize the same region with both human ACTB gene and a part of the POTE gene family. Although the nucleotide sequence of the actin part of the POTE gene family and human beta actin are similar, the POTE gene family is expressed in many cancers, but restricted to a few in normal reproductive tissues (prostate, testes, ovaries, and placenta) [28–30]. In this study, we determined gene expression from the PBMC of hyperlipidemia, CHD patients, and normal volunteers with no underlying disease. Therefore, no target for the POTE gene family could interfere in the present study. Similarly, some earlier studies used the ACTB primer from a similar region to our study. These primer sets were also predicted to amplify the POTE gene family [31, 32]. In addition our study using ACTB as a house keeping gene was in agreement with previous studies related to α − defensin gene expressions in other diseases [33–35] and other gene expression [17]. We suggest these ACTB primers were appropriate for normalizing the relative expression level of the DEFA1/DEFA3 gene.

DNA microarray analysis revealed that DEFA1/DEFA3 are expressed in both hyperlipidemia and CHD patients (Fig. 2). Our results showed that DEFA1/DEFA3 mRNA expression was not significantly different between these groups (*p* > 0.05; Fig. 3a), i.e., DEFA1/DEFA3 expression was similar between hyperlipidemia and CHD patients. Therefore, we suggest that DEFA1/DEFA3 may serve as a biomarker for CHD development in hyperlipidemia patients.

The highest HNP 1–3 concentrations were found in CHD patients (Fig. 3b), and differences were observed in HNP 1–3 levels between CHD and hyperlipidemia patients (*p* = 0.036), and healthy controls (*p* = 0.004). These elevated HNP 1–3 levels may be the consequence of the degranulation of neutrophils recruited from atherosclerotic plaques [2].

Our findings revealed a correlation between HNP 1–3 levels and TC (*r* = 0.530, *p* = 0.024) and LDL (*r* = 0.525 *p* =0.030) levels, but not TG (*r* = 0.525 *p* =0.030) or HDL (*r* = −0.870, *p* = 0.714; Fig. 4) levels. These observations are in line with a previous study on human patients [36] and an in vitro study [37]. Moreover, an earlier murine model, lacking HNP expression, suggests that HNP and LDL play equally important roles in the pathogenesis of vascular disease [38]. Collectively, these findings are in agreement with our results. CHD patients underwent coronary bypass grafting. Therefore, the TC, LDL and TG levels of these patients were significantly lower than those of hyperlipidemia patients, and similar to those of control patients. Interestingly, we observed that the TC and LDL levels decreased to normal ranges following administration of the appropriate lipid-lowering medication, while the HNP 1–3 levels remained elevated compared with the control and hyperlipidemia patients (Fig. 3b). We suggest that chronic neutrophil recruitment to atherosclerotic plaques in the vasculature of CHD patients was not inhibited by lipid-lowering drugs. Therefore, the highest HNP levels were found in the CHD group. Moreover, our findings on HNP expression provide new insight into CHD pathogenesis, which may affect future applications.

In contrast to a murine model [38], α-defensin in a porcine model reduced EC-dependent vasorelaxation. This effect is associated with increased superoxide radical production and decreased endothelial nitric oxide synthase (eNOS) expression in porcine coronary arteries [9].

Earlier studies have found discrepancies in HNP levels in sera [39–41], plasma [42] or biological fluids [41] of normal and various diseases including infectious diseases [41, 43, 44], metabolic diseases (e.g., diabetes) [45, 46], cardiovascular disease [2, 47], cancers of urinary bladder [42], colorectal cancer [40], colorectal adenoma [48] and colon carcinoma [39]. It has been reported that normal plasma levels of HNPs range from undetectable to 50–100 ng/ml. Similarly, our findings showed that normal HNPs’ levels ranged from undetectable to 10 ng/ml (Fig. 3b). Earlier studies have found that at the onset of bacterial infection and during nonbacterial infection, the mean HNP levels were 2- to 4-fold of those in healthy volunteers [43]. In bacterial meningitis, plasma HNP levels in sepsis patients ranged from 900 to 170,000 ng/ml compared with a mean of 42–53 ng/ml in the plasma of healthy controls [44].

We found that the increased levels of HNP 1–3 were moderately associated with DEFA1/DEFA3 expression (*r* = 0.536, *p* = 0.010). Previous studies have reported increased levels of HNPs in inflammatory diseases, indicating that HNPs play a critical role in the leukocyte-dominant proinflammatory responses that may contribute to cardiovascular disorders [49, 50] and bacterial meningitis [44]. Moreover, it has been reported that HNP deposits in skin biopsies are a strong independent predictor of coronary artery disease [41]. This suggested a link between PMN activation and progression of atherosclerosis, and provided a novel approach to assess the risk of coronary artery disease.

There is a correlation between HNP concentration and the number of blood PMNs in patients with inflammatory diseases [43]. Previous studies have reported a positive correlation between the peripheral PMN count and myocardial infarction [12]. However, we lacked data on the number of circulating neutrophils and the association between DEFA1/DEFA3 gene copy numbers and HNP 1–3 levels. Nevertheless, we assumed that the increased HNP levels, which are the main proteins in PMN granules, may correlate with PMN levels. In contrast, Nemeth et al. have reported that the copy number did not correlate with gene expression [45].

Quinn et al. have suggested a possible mechanism of action by which HNPs mediate cardiovascular disease. HNP 1–3 modulate the development of atherosclerosis by increasing the binding of LDL to the EC surface, promoting accumulation of LDL in the vasculature and inhibiting fibrinolytic activity on the surface of vascular cells accumulating in atherosclerotic plaques [2]. Moreover, HNPs form stable, multivalent complexes with LDL [37], both in solution and on the cell surface [37]. They also stimulate the binding of ^125^I-labeled LDL to human umbilical vein endothelial cells (HUVECs), smooth muscle cells and fibroblasts in a dose-dependent and saturable manner [37]. Additionally, it has been proposed that HNP-LDL complexes bind to heparin sulfate-containing proteoglycans (HSPG) [37]. The LDL receptor-related protein (LRP)/-2 macroglobulin receptor is a membrane protein of the LDL receptor (LDLR) superfamily [51] involved in atherogenesis [52, 53]. Increased expression of LRP has been demonstrated in vascular smooth muscle cells isolated from human atherosclerotic lesions [54]. LRP1 gene expression is also increased in blood mononuclear cells from patients with myocardial infarction [51]. An earlier study has demonstrated that HNPs directly bind LRP both in solution and on the surface of smooth muscle cells [55]. The structure of HNPs, with a hydrophobic and cationic site, is similar to many apolipoproteins that bind to LDLR family members and proteoglycans [37]. Hence, the ability of HNPs to modulate the catabolism of LDL may occur through similar mechanisms [37]. However, because many molecules (e.g., ApoE, thrombospondin, protein C, tPA, thrombin) are also ligands of LRP, the biological consequences of HNP and LRP binding remain to be investigated in the pathogenesis of atherosclerosis [51].

A previous study [29] used an *ApoE*^−*/−*^ mouse model, which is important for studying the mechanisms by which LDL, as a sole mediator, induces atherosclerosis. Murine PMNs lack HNPs, which raises the question of the importance of HNPs in the atherosclerotic pathology observed in *ApoE*^−*/−*^ mice. The study has demonstrated that the HNP-mediated inflammatory responses and the direct effects of LDL on the pathogenesis of cardiovascular diseases are equally important. PMN infiltration in chronic arterial inflammation, and the pivotal role of PMN contributing to atherogenesis was supported by a decrease in the atherosclerotic burden when PMNs were depleted in *ApoE*^*−/−*^ mice [29].

Our cross-sectional findings demonstrated increased expression of α-defensin during CHD development. Therefore, this could be indirectly interpreted as an important role for neutrophils in the development of atherosclerosis and consequently CHD. In contrast, our study indicated that DEFA1/DEFA3 mRNA expression and plasma HNP levels were consistent, hence they should be considered for further cross-sectional or larger cohort studies, including more randomized population groups, to elucidate the feasibility of predictive CHD biomarkers. We found a moderate correlation between HNP expression and disease development. This suggests that there may be more appropriate predictive markers that could lead to more reliable prediction of CHD. Additional studies are needed to validate whether additional genes (Table 2) are appropriate as inflammatory predictive markers for the risk of CHD development in Thai populations.

### Limitations

This study has some limitations. First, our sample size was small due to a limited budget. Statistical bias might occur. Further studies with a larger sample size and alternative simple techniques to detect markers are needed to confirm the current hypothesis. In addition, longitudinal studies are clearly needed to better define the importance of α-defensin expression. However, the hyperlipidemia and CHD groups significantly differ in age; thus, a longer observation period is needed for a cohort study. In this study we did not examine the α-defensin mRNA expression and plasma HNP 1–3 levels in medical treatment hyperlipidemia and CHD patients, which would provide more evidences to support our hypothesis. The effect of drugs on decreasing cholesterol levels is a very interesting factor, but was considered excessively complex for inclusion in the present study. Various lipid-lowering drugs are used, with different modes of action. Further studies with larger sample sizes would be more appropriate to assess the effects of these drugs on the development of CHD. Furthermore, the absence of neutrophil numbers and DEFA1/DEFA3 copy number is another limitation.

## Conclusions

Taken together, our findings suggest that hyperlipidemia patients with elevated TC and/or LDL levels in combination with increased α-defensin mRNA expression and/or elevated plasma HNP levels may be at risk of developing CHD.

## Abbreviations

CHD, coronary heart disease; ELISA, enzyme-linked immunosorbent assay; HDL, high-density lipoprotein; HNP 1–3, human neutrophil peptides 1–3; LDL, low-density lipoprotein; PBMC, peripheral blood mononuclear cells; qRT-PCR, quantitative reverse transcription-polymerase chain reaction; TC, total cholesterol; TG, triglyceride; α-defensin, alpha-defensin.
